# Laparoscopic pancreaticoduodenectomy in A-92-older Chinese patient for cancer of head of the pancreas: A Case report

**DOI:** 10.1097/MD.0000000000005962

**Published:** 2017-01-20

**Authors:** Jiayu Zhou, Chang Xin, Tao Xia, Yiping Mou, Xiaowu Xu, Renchao Zhang, Yucheng Zhou, Weiwei Jin, Chao Lu

**Affiliations:** a Department of General Surgery, School of Medicine, Zhejiang University; b Department of General Surgery, Key Laboratory of Gastroenterology of Zhejiang Province, Zhejiang Provincial People's Hospital, Hangzhou; c Department of Hepatobiliary Surgery, Yinzhou Hospital Affiliated to Medical School of Ningbo University, Ningbo, Zhejiang Province, China.

**Keywords:** elderly, laparoscopic pancreaticoduodenectomy, minimally invasive surgery, pancreatic cancer

## Abstract

**Introduction:**

Laparoscopic pancreaticoduodenectomy (LPD) is one of the most complex gastrointestinal procedures performed in laparoscopic abdominal surgery. However, the concern for elderly undergoing LPD remains. To the best of our knowledge, there are no reports describing LPD for A-92-older patient. This study aimed to share the experience of a tertiary pancreatic center and confirm the safety, feasibility of LPD for the elderly.

**Method:**

The patient had complained of 6-months history of abdominal discomfort and progressive jaundice. Abdominal computed tomography CT/MR imaging revealed a 3 × 3 cm solid hypovascular mass in the head of the pancreas. LPD was successfully performed after multidisciplinary team (MDT). Operation time was 450 minutes, and blood loss was 120 mL. Histological examination of the resected specimen confirmed the diagnosis of pancreatic ductal adenocarcinoma (PDAC).

**Outcomes:**

The patient was discharged on POD13 following an uneventful postoperative period. She was followed up 4 months without any sign of recurrence.

**Conclusion:**

LPD can be performed safely in patients of any age who are fit for surgery in specialist centers.

## 1 Introduction

The incidence of pancreatic cancer increases with age and is therefore an important disease for the elderly. Surgery is the only curative treatment currently available for pancreatic ductal adenocarcinoma. However, advanced age is frequently considered as a limitation to open pancreatic surgery.^[1]^ Over the last 2 decades, many breakthroughs in technological innovation and surgical strategies have made pancreatic surgical procedures safe including laparoscopic pancreaticoduodenectomy (LPD).^[2]^

LPD introduced in 1990s involves the same techniques as its open counterpart including dissection of the duodenum and the head of the pancreas, and reconstruction of the gastrointestinal tract.^[3]^ Many published literatures have demonstrated the advantages of this procedure such as shorter hospital stay and quicker recovery.^[4]^ With regards to oncologic outcomes, there was no definite conclusion. Therefore, according to these, some high volume centers performed LPD in the elderly patients and reported mortality rate of this operation has improved from 30% previously to 10%.^[5]^ In our institution, we have performed LPD as safely in elderly patients as in younger ones. No different in mortality rate and sever complications were found. The significant improvement has encouraged us to carry out LPD in A-92-older patient.

To the best of our knowledge, LPD in age over 90 was not reported.^[6]^ In this study, we present a successful case LPD without morbidity through multidisciplinary team (MDT) meetings and the perioperative management.

## 2 Case report

**Figure 1:**
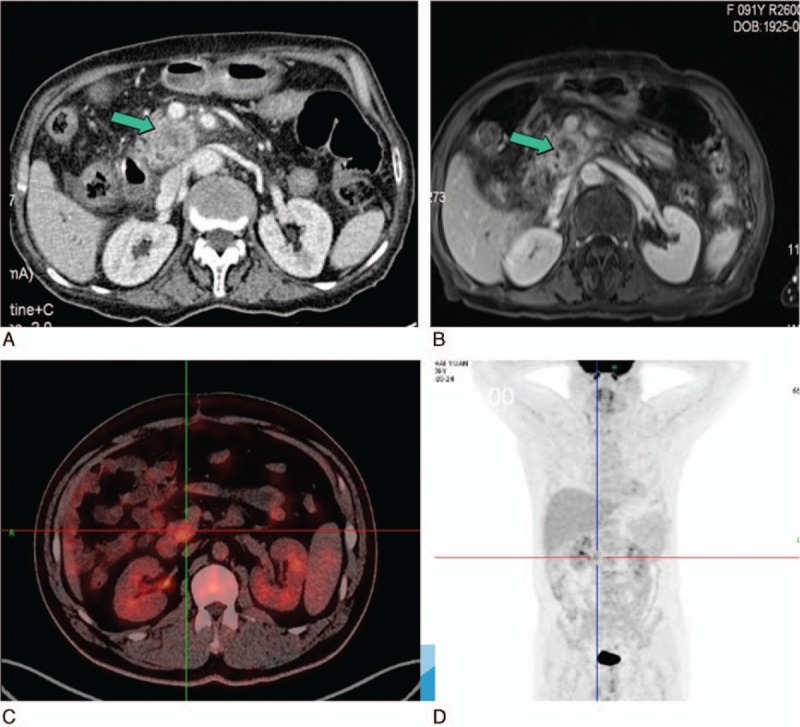
(A) Abdominal contrast-enhanced CT and (B) contrast-enhanced MRI showed a 3 × 3 cm solid hypovascular tumor in the head of the pancreas (green arrow). (C, D) PET-CT showed a mass with SUVmax 11.6 located in the pancreatic head and no distant organ metastasis. CT = computed tomography, MRI = magnetic imaging resonance, PET = positron emission tomography.

A 92-year-old female patient was referred to our hospital on May 9, 2016 with a pancreatic tumor. She had complained of 6-months history of abdominal discomfort and progressive jaundice. Her past medical history was unremarkable. There were no significant findings on physical examination with the exception of severe malnutrition. Height was 148 cm and weight was 38.5 kg. Laboratory findings were as follows: lymphocyte count, 1590/mm^3^; total bilirubin, direct bilirubin, and albumin, 3.03 g/dL; and amylase and IgG-4 were within the normal range. The serum level of carbohydrate antigen (CA)19-9 was 235 μmol/L, and carcinoembryonic antigen (CEA), CA72-4, and CA12-5 were all normal. Abdominal contrast-enhanced computed tomography (CT) scan and magnetic resonance (MR) imaging revealed a 3 × 3 cm solid hypovascular mass in the head of the pancreas (Fig. 1A, B). The common bile duct and the main pancreatic duct on the distal side of the mass were dilated 12 and 7 mm, respectively. PET-CT revealed that a mass with SUVmax 11.6 in the head of pancreas. There was no evidence of lymph node metastasis, peritoneal dissemination, or distant organ metastasis (Fig. 1C, D).

**Figure 2:**
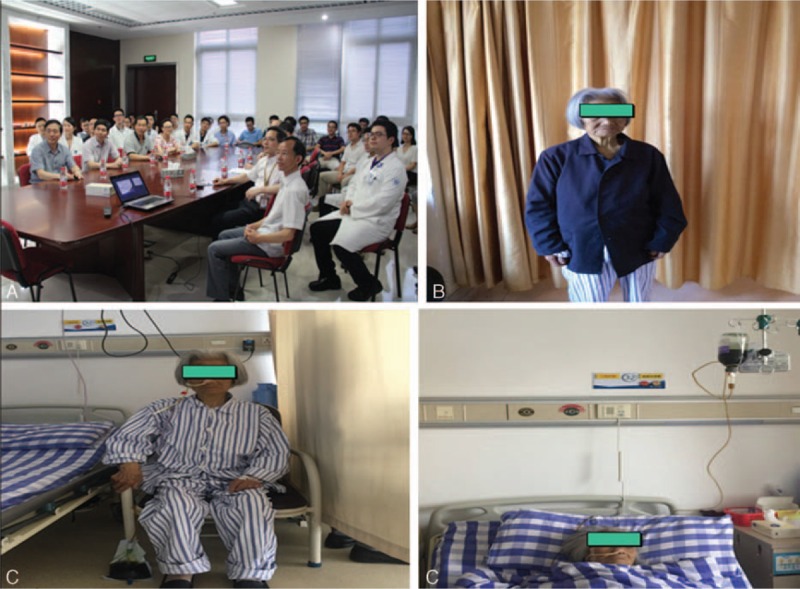
(A) Multidisciplinary team meeting, (B) a-92-older patient with malnutrition, and (C, D) the status of the patient treated by enteral nutrition and parenteral nutrition.

MDT meeting consisted of surgeons, physicians, clinical and medical oncologists, radiologists, pathologists, and clinical nurse specialists (CNSs) were organized to make clinical decisions (Fig. 2). There are 3 results of discussions. First, our tentative diagnosis was resectable pancreatic adenocarcinoma greater than 2.0 cm in size, T2N0M0 clinical stage IB on the TNM classification of the International Union Against Cancer (UICC). Second, the patient was obstructive jaundice and severe malnutrition. ASA was II-stage. Prognostic nutritional index was 38.2. These caused surgical contraindication. Third, percutaneous transhepaticcholangial drainage (PTCD) was used to relieve jaundice and bile reinfusion (Fig. 2). Malnutrition was improved by enteral nutrition and parenteral nutrition 2 weeks later. Prognostic nutritional index increased by 47.45 and TB reduced to 107 μmmol/L. Meanwhile, breathing exercise was strengthened during this period.

**Figure 3:**
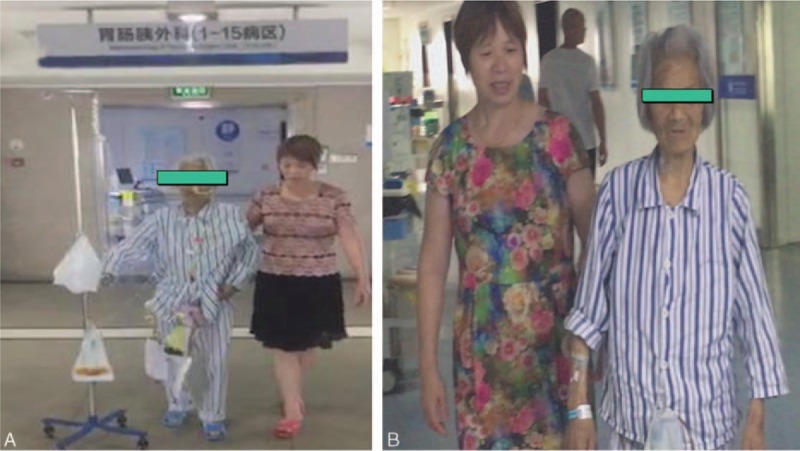
(A) On postoperative day (POD)2, the patient achieved out-of-bed activity. (B) On POD13, she was discharged.

**Figure 4:**
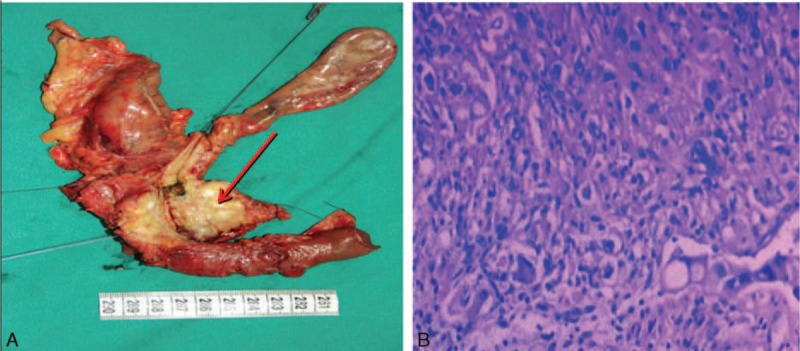
(A) Gross appearance of resected specimen: the lesion located in the head of the pancreas (red arrow), (B) histopathology of the pancreatic ductal adenocarcinoma (PDAC) (H&E × 100). The tumor represented moderately differentiated ductal adenocarcinoma.

The informed consent was signed and LPD was successfully performed. Intraoperative frozen section confirmed pancreatic ductal adenocarcinoma (PDAC) without peritoneal metastasis, and R0 resection was achieved. Operation time was 450 minutes, and blood loss was 120 mL. On the 1st postoperative day (POD1), the nurse assists the patient move her limbs on the bed to prevent deep venous thrombosis. On POD2, the nasogastric tuber was removed and the patient achieved out-of-bed activity (Fig. 3A). She started liquid diet on POD3 and CT was performed on POD7 without abdominal effusion fluids. On POD8, drainage tubes were removed and she was discharged on POD13 (Fig. 3B). No pancreatic fistula, pulmonary complication, or bile leakage was found during the hospital stay. Histopathological and immunohistochemical examination of the resected specimen revealed that it is the moderately differentiated pancreatic duct adenocarcinoma without lymph nodes metastasis (Fig. 4).

After operation, the patient refused to receive chemotherapy. Four months later, we followed up the patient. Abdominal CT was conducted to evaluate the regional recurrence. No abnormity was found. The tumor markers including Ca19-9, CEA, Ca125 were in normal range.

## 3 Operative technique

**Figure 5:**
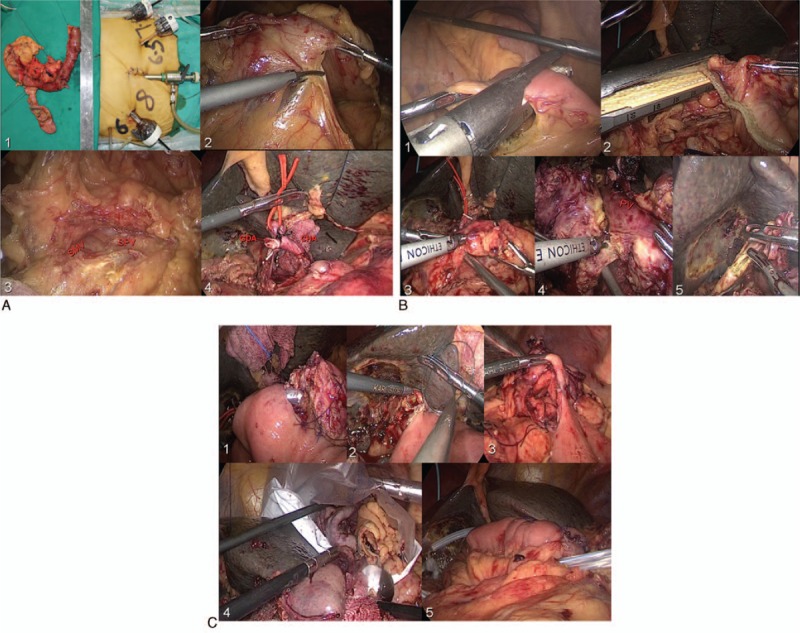
(A) 1 – Showing 5 trocars in a V-shape; 2, 3 – dividing the gastrocolic ligament, exposing the pancreas, SMV, and SPV; and 4 – ligating the GDA. (B) 1, 2 – The jejunum and stomach dissection (linear stapler); 3 – pancreatic neck division; 4 – dissecting the uncinate process of pancreas; and 5 – the bile duct division. (C) 1 – The end-to-side pancreaticojejunostomy (duct-to-mucosa); 2 – the end-to-side choledochojejunostomy; 3 – the gastrojejunostomy; 4 – the specimen removed; and 5 – placing 2 drainage tubes. GDA = gastroduodenal artery, SMV = superior mesenteric vein, SPV = splenetic vein.

Port placement adopted 5 trocars in a V-shape depicted in Fig. 5A. The surgeon stands in the right hand and the 1st assistant in the left side.

### 3.1 Step 1-resection

Dividing the gastrocolic ligament, exposing the pancreas (Fig. 5A, B)

Excising the right gastroepiploic vessels, ligating the gastroduodenal artery(GDA)

Establishing a portal vein tunnel

Kocher maneuver

Jejunum, stomach dissection (linear stapler): bile duct division (scissors) Pancreatic neck division (Harmonic scalpel with scissors at pancreatic duct)

The uncinate process is dissected along the adventitia of the superior mesenteric artery

The specimen is placed in a bag for retrieval

### 3.2 Step 2-reconstruction

An end-to-side pancreaticojejunostomy (duct-to-mucosa) with stent (Fig. 5C)

An end-to-side choledochojejunostomy using the 4-0 V-LOC suture is subsequently fashioned

The gastrojejunostomy is performed antecolic using a stapled technique.

## 4 Discussion

The aging population in the world is growing at an unprecedented rate.^[7]^ In the year 2010 the worldwide incidence of PDAC was 315,600 cases.^[8]^ Sixty percent of them were over the age of 65. Radical surgical resection for select individuals with PADC is the only potentially curative treatment. LPD is a complex procedure which often operated by experienced surgeon in high volume institutions.^[9]^ Until now, What is not clear is that patients aged 75 years and older can benefit from LPD for PADC.^[10]^

MDT meeting offer clinical decision making through the multidisciplinary collaboration. From the July 2015, MDT was organized to treat the PDAC in our center. In this case, it played an indispensable role of the Clinical medical decision-making. Severe malnutrition considered to be the surgical contraindication was improved obviously after 2-week nutrition support. What is more, MDT not only delivered the patient's condition to her relations but also was contribute to doctor–patient communication, especially in China. The anesthetist participating in MDT meeting conduced to intraoperative management.

Minimally invasive surgery not only represents mini-incision and aesthetics but also reduces the morbidity and recovery time.^[11]^ Previously published articles reported that LPD have lower intraoperative blood loss, transfusion, intensive unit stay, hospital stay, and postoperative complications when compared with OPD. In this case, the advantage of LPD had fully reflected. No postoperative pulmonary complication (PPC) which is a reliable risk factor for mortality occurred. Two potential factors may be responsible for the satisfied result.^[12]^ First of all, LPD using only 5 trocars instead of traditional straight long wound by OPD lead to less abdominal pain reducing inflammatory reaction. As it reported previously the degree of the postoperative inflammatory response is smaller following laparoscopic procedures compared with open surgery. Meanwhile, laparoscopic surgery has little impact on respiratory movement due to small incision. Second, intraoperative blood loss less than OPD was 120 mL and no transfusion was found reducing the risks of PPC.^[13]^

LPD combined with enhanced recovery after surgery offered the technological base for the patient faster recovery. In this study, she took off-bed activity on POD2 and liquid diet on POD3.

What is more, postoperative hemorrhage, pancreatic fistula, bile leakage, and delayed gastric emptying were not found before her discharge within 13 days.^[14]^ Of course, it is ascribed to not only carefully postoperative management but also the skilled surgeon.^[15]^ In our center, LPD operated by doctor Mou was performed in 180 cases including 12 elderly patients aged 80 and older. Accumulated experience including surgical skills and intrapostoperative management provided us confidence to complete this procedure. The following 3 main experiences were concluded. First, 5-hole approach was used. It fits Chinese body and is comfortable for the surgeon and the 1st assistant to perform the surgery. Second, harmonic scalpel was regularly applied in our center. Finally, LPD has longer operation time, so intraoperative warming is important to prevent coagulation disorders because of hypothermia.

Many articles suggested performance status was considered as a clinically relevant risk factor for short-term outcome. In this study, Eastern Cooperative Oncology Group performance status (PS) of the patient was good and the score of Eastern Cooperative Oncology Group-PS was one. The patient was discharged after surgery following an uneventful postoperative period. Therefore, the question needs to be asked if performance status better than age can also be used to guide surgical treatment in the elder patients. More studies were needed to confirm this question.

In conclusion, age alone should not be a contraindication for LPD in advanced patients. Our study indicates that LPD is feasible in elderly patients and conduced to rapid postoperative recovery in high-volume centers. It also encourages the surgeons to consider LPD as a therapy in elderly adults. However, the long-term outcome of this surgery needs to be confirmed. Many studies with a large number of cases are needed to provide more reliable information.
